# *Staphylococcus aureus* Carotenoids Modulate the Thermotropic Phase Behavior of Model Systems That Mimic Its Membrane Composition

**DOI:** 10.3390/membranes12100945

**Published:** 2022-09-28

**Authors:** Marcela Manrique-Moreno, Małgorzata Jemioła-Rzemińska, Jessica Múnera-Jaramillo, Gerson-Dirceu López, Elizabeth Suesca, Chad Leidy, Kazimierz Strzałka

**Affiliations:** 1 Chemistry Institute, Faculty of Exact and Natural Sciences, University of Antioquia, Medellin 050010, Colombia; 2 Faculty of Biochemistry, Biophysics and Biotechnology, Jagiellonian University, 30-392 Krakow, Poland; 3 Malopolska Centre of Biotechnology, Jagiellonian University, 30-392 Krakow, Poland; 4 Laboratory of Advanced Analytical Techniques in Natural Products (LATNAP), Chemistry Department, Universidad de los Andes, Bogotá 111711, Colombia; 5 Biophysics Group, Department of Physics, Universidad de los Andes, Bogotá 111711, Colombia

**Keywords:** *Staphylococcus aureus (S. aureus)*, carotenoids, bacterial membrane, differential scanning calorimetry, infrared spectroscopy

## Abstract

*Staphylococcus aureus* (*S. aureus*) is a pathogenic gram-positive bacterium that normally resides in the skin and nose of the human body. It is subject to fluctuations in environmental conditions that may affect the integrity of the membrane. *S. aureus* produces carotenoids, which act as antioxidants. However, these carotenoids have also been implicated in modulating the biophysical properties of the membrane. Here, we investigate how carotenoids modulate the thermotropic phase behavior of model systems that mimic the phospholipid composition of *S. aureus*. We found that carotenoids depress the main phase transition of DMPG and CL, indicating that they strongly affect cooperativity of membrane lipids in their gel phase. In addition, carotenoids modulate the phase behavior of mixtures of DMPG and CL, indicating that they may play a role in modulation of lipid domain formation in *S. aureus* membranes.

## 1. Introduction

*Staphylococcus aureus* (*S. aureus*) is a gram-positive bacterium characterized by its common implication in infections related to cystic fibrosis, endocarditis, septic arthritis, and medical implanted catheters. These infections range from minor skin and wound diseases to life-threatening sepsis [1]. *S. aureus* is considered an opportunistic microorganism since it normally colonizes healthy human populations and is found safely in different sites such as the skin and the nose channel, becoming a risk factor if these tissues are compromised or the immune response is impaired. This pathogen also has the ability to live for extended periods on different surfaces such as walls within surgery rooms and surgical instruments, in addition to its ability to reside in the interior of different cell types within the human body during chronic infections [2]. Interestingly, this occurs in the absence of the ability to sporulate, which is normally used by other bacteria as a way to tolerate extreme conditions. It is clear that *S. aureus* is a highly versatile bacterium that can survive and thrive in different environmental states. In particular, it has been suggested that *S. aureus* has the ability to live in extreme conditions, especially at low temperatures, due to its ability to regulate the lipid composition of its cell membrane [3].

This astonishing versatility makes it particularly intriguing to understand the different biophysical aspects that make *S. aureus* so adaptable. It is well-established that one of the biological macrostructures that requires more attention is the plasma membrane. In particular, its ability to regulate different biophysical aspects of the bilayer through variations in lipid composition has recently drawn attention. Recent publications have explored the role that different lipid components play in the resistance of *S. aureus* to different stress factors [4]. For example, regulation of the proportion of branched to unsaturated lipids has been shown to play a role in the homeoviscous response. Additionally, the synthesis of carotenoids has been associated with resistance to antimicrobial peptide activity. The membranes of *S. aureus* are mostly composed of phosphatidylglycerol lipids with varying acyl chains. These acyl chains are mainly saturated or branched in iso- and anteiso- conformations. *S. aureus* does not synthesize unsaturated lipids and, in general, does not contain any unless exposed to media enriched in these lipids. Another major phospholipid is cardiolipin, which accounts for around 20% of the total lipid content [5]. The presence of this four-chained phospholipid has been associated with greater tolerance to osmotic stress and resistance to antimicrobial peptide activity [6,7]. Carotenoids are another major component that gives *S. aureus* its characteristic golden color. These pigments are isoprenoid derivatives that have been associated with antioxidant, anti-inflammatory, and photoprotective activities, among others, because of their ability to delocalize charges and radicals in their conjugated double-bond chains [8]. Carotenoids are associated with tolerance to oxidative stress and regulation of the mechanical properties of the bacterial membrane [9,10,11]. Recently, a composition of total carotenoids from *S. aureus* at 24 h of growth has been reported, which was made up of two identified compounds: 4,4’-diaponeuresporenoic acid and staphyloxanthin (STX) (including its homologues) [12,13], both related to the increase in the virulence and bacterial fitness of *S. aureus* [14,15]. In a previous publication, it has been shown that carotenoid production depends on growth conditions, where carotenoids are synthesized in large quantities in the stationary phase but are not present in the exponential phase or in biofilms grown under flow conditions in the dark [11]. Carotenoids of *S. aureus* have been shown to influence membrane structure, appearing to increase the order of the acyl chain, as measured by 1,6-diphenyl-1,3,5-hexatriene (DPH) anisotropy and Laurdan generalized polarization [11]. This increase in the order of the acyl chain has been associated with increased resistance to antimicrobial peptides such as daptomycin and magainin [16,17,18]. The high concentration of carotenoids of *S. aureus* produces changes in the physicochemical properties of the membrane [19], preventing the insertion of daptomycin, which blocks the ability of the antibiotic to generate pores in the bacterial membrane [20]. STX and related carotenoids from *S. aureus* have been shown to enable the survival of the microorganism in presence of oxidation processes due to reactive oxygen, nitrogen, and chlorine species [10,13]. STX and its derivatives have been reported to have the ability to form functional membrane microdomains, leading to resistance to antibiotic and virulence in bacteria [21]. Furthermore, a comparative study between carotenoid- and non-carotenoid-producing *S. aureus* cells showed that pigmented cells have the best tolerance to high temperatures when cells are grown to the stationary phase [22], hence the importance of understanding the biophysical implications of these carotenoids in the membrane of *S. aureus*.

The purpose of this work was to evaluate the function of *S. aureus* carotenoids in model membrane systems containing 1,2-dimyristroyl-sn-glycero-3-phosphatidylglycerol (DMPG) and 1′,3′-bis[1,2-dimyristoyl-sn-glycero-3-phospho]-glycerol (CL) through differential scanning calorimetry (DSC) and Fourier transformed infrared spectroscopy (FT-IR) experiments. Here, we focus on a representative saturated acyl chain of 14 carbons for both DMPG and CL. We report mainly on the effect of carotenoids on the gel (L_β_) to liquid–crystalline (L_α_) phase transition of these two lipids as a representative membrane for *S. aureus*, focusing on the shifts in the position of the phase transition temperature and changes in the cooperativity of the transition. We also investigated how the presence of carotenoids influences the miscibility of DMPG:CL mixtures in order to elucidate aspects related to the regulation of domain formation in *S. aureus* membranes. From a different perspective, the study of the thermotropic behavior of *S. aureus* membranes has led to the identification of different characteristic thermal events in the live cell membrane that are clearly sensitive to lipid composition.

## 2. Materials and Methods

### 2.1. Chemical Reagents

The required 1,2-dimyristoyl-sn-glycero-3-phosphoglycerol sodium salt (DMPG, Lot. 140PG-167) and 1′,3′-bis[1,2-dimyristoleoyl-sn-glycero-3-phospho]-glycerol sodium salt (cardiolipin, CL, Lot. 750332P-200MG-A-030) were purchased from Avanti Polar Lipids (Alabaster, AL, USA). HPLC-grade methanol, ethyl acetate, and chloroform were purchased from Honeywell (Detroit, MI, USA) and J.T. Baker (Palo Alto, CA, USA) respectively. NaCl Reagent Plus (>99%) and butylated hydroxytoluene (BHT) were purchased from Sigma-Aldrich (St. Louis, MO, USA). Luria–Bertani (LB) medium was prepared with NaCl (ACS, J.T. Baker, USA), tryptone (OXOIO, Basigstoke, Hampshire, UK), and a yeast extract (Dibico, México D.F., México). Deionized water was obtained from a water purification system: Heal Force Smart-Mini (Shangai, China) and (Thermo Scientific, Waltham, MA, USA).

### 2.2. Bacterial Cultures

A clinical methicillin-susceptible *S. aureus* strain was used, whose carotenoid chemical composition and its biophysical properties have been previously described [11,12]. A single colony of the strain was grown in 10 mL of LB medium at 37 °C overnight (16 h) with constant agitation (250 rpm). LB medium contained, per liter, 10 g of NaCl, 10 g of tryptone, and 5 g of the yeast extract. Next, the cells were diluted (1:1000) in fresh LB medium and cultivated for 24 h. Then, the cell pellet was obtained by centrifugation at 7300× *g* at 4 °C for 10 min (Thermo Scientific, USA), frozen at −80 °C, and lyophilized for 24 h (LABCONCO, Kansas City, MO, USA).

### 2.3. Carotenoid Extraction

The extraction of carotenoids was achieved using a method previously reported by López et al., with some modifications [12]. A gram of freeze-dried and grounded cells (with mortar and pestle) was weighed in a Falcon tube and dissolved in 20 mL of methanol (MeOH) containing BHT at 0.1%, *w*/*v*; then, 20 glass beads were added and vortex-mixed for 5 min. After carotenoids dissolved in methanol were obtained by centrifugation at 7300× *g* for 10 min at 4 °C, the extraction was repeated twice with 10 mL of MeOH–BHT (0.1%, *w*/*v*). Methanolic phases were combined, shaken with the mixture of ethyl acetate and 1.7M NaCl (1:3 *v*/*v*), and centrifuged at 7300× *g* for 15 min at 4 °C. The upper organic phase was evaporated in a rotary evaporator with 35 °C in water bath. For protein removal, the extract was solubilized in 20 mL of mix CHCl_3_:MeOH (2:1) and transferred to a borosilicate glass tube with a phenolic cap and polytetrafluoroethylene (PTFE)-faced rubber liner; then, 7 mL of 1.7 M NaCl was added, and the solution was shaken. Upon centrifuging at 630× *g* for 15 min at 4 °C, phase separation was achieved, and the lower CHCl_3_ phase containing carotenoids was collected. Then, the organic phase was dried with anhydrous Na_2_SO_4_, transferred to an amber tube, and finally dried with a CentriVap refrigerated vacuum concentrator (LABCONCO, Kansas City, MO, USA). The composition of the extract was confirmed using LC-MS/MS method with the conditions previously reported [12].

### 2.4. Differential Scanning Calorimetry (DSC)

For calorimetric experiments, appropriate amounts of DMPG and CL were dissolved in chloroform:methanol (1:1) and pure chloroform, respectively, to prepare a stock solution. Volumes of each lipid were deposited in a glass tube in order to obtain a 1 mM final phospholipid concentration of DMPG:CL (80:20). The solvent was dried under a stream of nitrogen, and the traces were removed by keeping the sample under reduced pressure (about 13.3 Pa) for 30 min. The dried films were hydrated in buffer (10 mM HEPES, 500 mM NaCl, 1 mM EDTA, pH 7.4). Multilamellar vesicles (MLV) were formed by vortexing the samples above the main phase transition temperature of the lipids for at least 15 min. Carotenoids stock solution was prepared in chloroform and added in various volumes to the lipids dissolved in chloroform, depending on the weight-to-weight ratio of the mixture. The films were then hydrated in a buffer in the same way as described above. DSC measurements were performed on a Nano DSC device (TA Instruments, New Castle, PA, USA). The sample cell was filled with 400 µL of MLV suspension and an equal volume of buffer was used as a reference. Cells were sealed and equilibrated for about 10 min at the starting temperature. Heating/cooling rates were 1 °C per minute and the scans were recorded within a range of 5 to 60 °C. Heating scans were carried out first. The reference scan was subtracted from the sample scan. Each dataset was analyzed, and the values of thermodynamical parameters were calculated using a software package supplied by TA Instruments. The transition temperature, T_m_, determined where the heat capacity, Cp, reached its maximum value. The value of the calorimetric enthalpy (ΔH) for the phase transition was determined by integrating the area under the peak. From these values, the entropy of the phase transition was determined by ΔS = ΔH/T_m_. At least three independently prepared samples were measured to verify the reproducibility of the DSC experiments. The accuracy was ±0.1 °C for the main phase transition temperature and ±1 kJ mol^−1^ for the main phase transition enthalpy.

### 2.5. Infrared Spectroscopy Experiments

Solid-supported lipid bilayers were prepared in situ on a BioATR II cell. The unit was integrated to a Tensor II spectrometer (Bruker Optics, Ettlingen, Germany) with a liquid-nitrogen MCT detector with a spectral resolution of 4 cm^−1^ and 120 scans per spectrum. The desired temperature was set by a Huber Ministat 125 computer-controlled circulating water bath (Huber, Offenburg, Germany). First, the background was taken using HEPES buffer 20 mM, 500 mM NaCl, and 1 mM EDTA in the same temperature range. Subsequently, for coating the silicon crystal, stock solutions of pure DMPG, CL, DMPG:CL (80:20), and DMPG:CL with carotenoids were dissolved in chloroform. The cell was filled with 20 µL of a 20 mM lipid stock solution, and the chloroform was evaporated, resulting in a lipid film. For in situ measurements, the cell was afterwards filled with 20 µL of buffer or peptide solution and incubated over the phase transition temperature for 10 min. To determine the position of the vibrational band in the range of the second derivative of the spectra, all the absorbance spectra were cut in the 2970–2820 cm^−1^ range, shifted to a zero baseline, and the peak-picking function included in OPUS software was used. The results were plotted as a function of the temperature. To determine the T_m_ of the lipids, the curve was fitted according to the Boltzmann model to calculate the inflection point of the obtained thermal transition curves using OriginPro 8.0 software (OriginLab Corporation, USA).

## 3. Results

### 3.1. Differential Scanning Calorimetry (DSC) of DMPG:CL Mixtures in the Presence of Carotenoids from S. aureus

**Figure 1:**
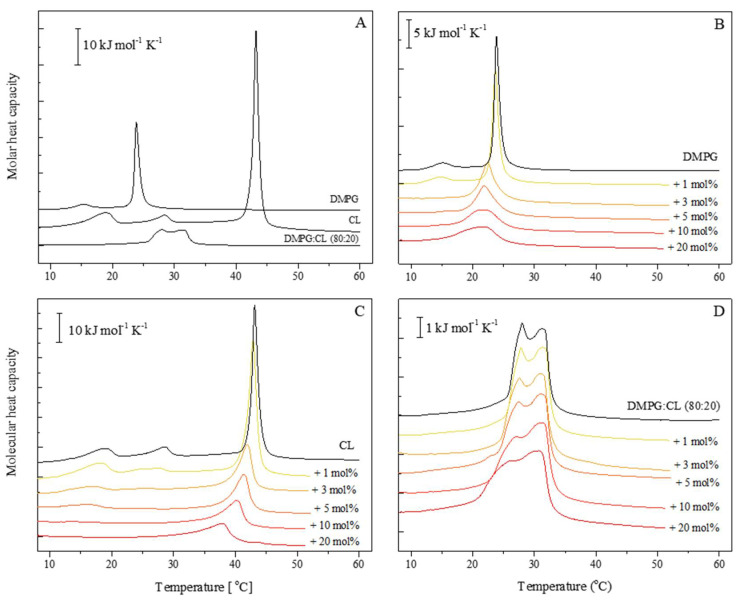
Representative DSC heating curves obtained for multilamellar liposomes of (**A**) DMPG, CL and their mixture DMPG:CL (80:20), and total carotenoid extract incubated with (**B**) DMPG, (**C**) CL, and (**D**) mixture DMPG:CL (80:20).

Calorimetric experiments were performed to determine the effect of carotenoids on the *S. aureus* membranes. Phosphatidylglycerol (PG) is the most abundant lipid (80%) in the outer monolayer and CL is a minor component (20%) [5,23,24]. Thermograms of the MLV made of DMPG, CL, and DMPG:CL (80:20) are summarized in Figure 1A. The thermogram of pure DMPG liposomes shows two endothermic peaks; the small peak at a lower temperature (15.03 °C) corresponds to the transition from gel phase (L_β_) into the ripple phase (P_β′_), also called a pretransition, and the large peak at a higher temperature (23.86 °C) corresponds to the main phase transition with a ΔH = 29.8 kJ mol^−1^. The P_β′_ is one of the gel phases that occur due to structural constraints between the packing characteristics of the two acyl chains and the head groups [25]. Fully hydrated CL liposomes show three endothermic transitions, in order of increasing temperature: the subtransition (14–18 °C), pretransition (25–30 °C), and the main transition temperature (approximately 43 °C) with a ΔH = 81.93 kJ mol^−1^. These results are consistent with the DSC thermogram reported by Lewis et al. in 2007 [26]. The thermogram of the mixture DMPG:CL (80:20) shows a heating curve composed of two wide and unresolved peaks, one at 28.10 °C (peak #1) and another at 31.27 °C (peak #2).

The results of the effect of carotenoids on the liposomes from DMPG and CL are presented in Figure 1B,C, respectively. Carotenoids are able to alter the cooperativity of the phase transition of both phospholipids in a concentration-dependent manner. Even at the lowest concentration evaluated (1 mol%), there is a shift of the T_m_ to lower values for both lipids, while the pretransition is still detectable for both lipid systems. However, at a concentration of 3 mol%, the metastable ripple phase disappeared and a progressive, slight decrease in T_m_ is observed. Furthermore, the enthalpy of the main phase transition decreased in comparison with the control by about 20 and 50% for 5 mol% carotenoids in the case of DMPG and CL, respectively. Higher concentrations of the carotenoids induced a pronounced broadening and lowering of the peaks in the thermograms obtained during heating of DMPG and CL liposomes.

**Figure 2:**
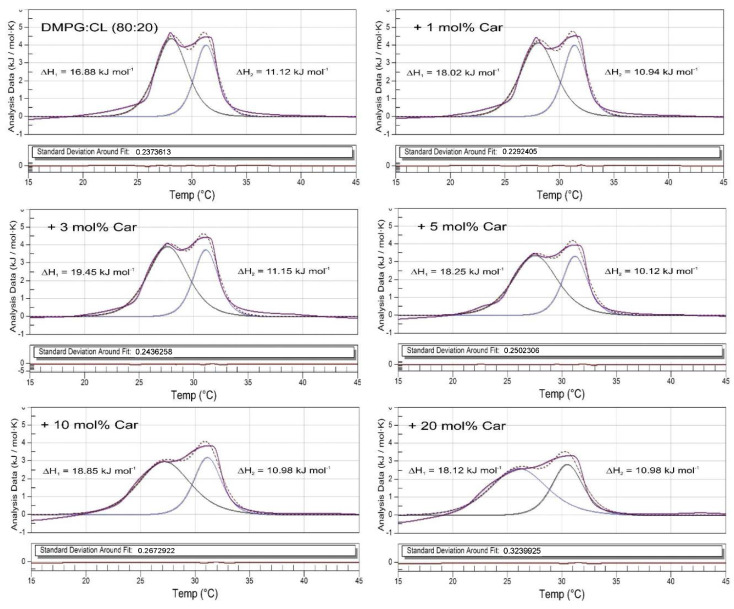
Deconvolution peaks of representative DSC heating curves obtained for multilamellar liposomes prepared from DMPG and CL mixture DMPG:CL (80:20).

**Figure 3:**
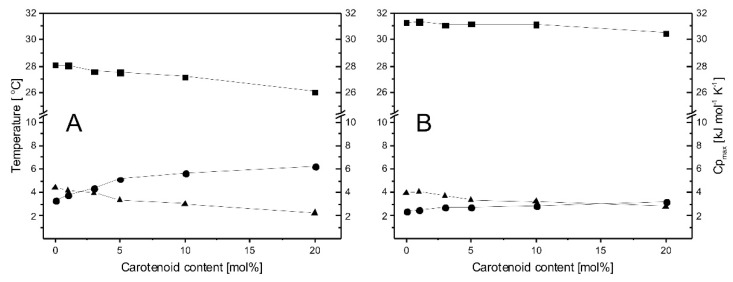
Plots of phase-transition temperatures T (■), full-width at half-maximum ΔT_1/2_ (●), and peak height Cp_max_ (▲) of deconvoluted peaks from representative DSC heating curves obtained for multilamellar liposomes prepared from DMPG and CL mixture (DMPG:CL, 80:20) as a function of carotenoid content. Sets of data for peak #1 and peak #2 are shown in panel (**A**) and (**B**), respectively.

The DSC heating curves obtained for DMPG:CL (80:20) liposomes upon addition of increasing amounts of carotenoids are presented in Figure 1D. In contrast to single-lipid liposomes, the DMPG:CL system is less susceptible to the presence of *S. aureus* carotenoids. Moreover, the observed modifications of thermotropic phase behavior of the lipid bilayers are not so concentration-dependent. However, since two peaks are present in the thermogram of the lipid mixture, a deconvolution procedure was performed to check if these two peaks are affected differently by the presence of carotenoids (Figure 2). Although there was a small change in the ΔH of peak #1, the area under peak #2 remained almost unaffected. Nevertheless, at higher concentrations of carotenoids, it is evident that peak #1 undergoes a greater broadening effect than peak #2. Thermodynamic parameters of the pretransition and main phase transition of DMPC and CL are presented in the Supplementary Materials (see Tables S1 and S2). The parameters of deconvoluted peaks from representative DSC heating curves obtained for multilamellar liposomes prepared from DMPG and CL mixture (DMPG:CL, 80:20) are presented in Table 1. Additionally, the dependence of phase-transition temperatures (T_m_), full-width at half-maximum (ΔT_1/2_), and peak height (Cp_max_) of deconvoluted peaks on the carotenoid content has been shown in Figure 3.

### 3.2. Phase-Transition Measurements by Infrared Spectroscopy

**Figure 4:**
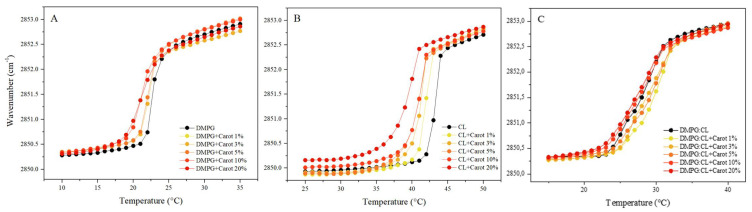
Peak positions of the ν_s_CH_2_ vibration bands of the methylene groups as a function of temperature of total carotenoid extract incubated with (**A**) DMPG, (**B**) CL, and (**C**) DMPG:CL (80:20).

The FT-IR technique has been extensively used to follow the methylene symmetric stretching vibrational mode (ν_s_CH_2_) as a function of temperature. It is known that this vibrational mode is sensitive to changes in the conformational order of lipid hydrocarbon chains and therefore it can be used to monitor the progress of lipid–gel/liquid–crystalline phase-transitions. The symmetric methylene stretching mode has different values in each phase of the lipids; in the gel phase, *ν*_s_CH_2_ lies at 2850 cm^−1^, and in the liquid crystalline phase, it is around 2852 cm^−1^ to 2853 cm^−1^ [27,28,29]. Depending on the length of the acyl chains [30] and the headgroups [31] of the phospholipids, T_m_ has a characteristic value. Figure 4A,B show the temperature dependence of the wavenumber values of the peak positions of the pure DMPG and CL lipids in the presence of different concentrations of carotenoids.

The interaction of carotenoids with both lipids resulted in a shift of T_m_ to lower values, as well as fluidization of the lipid systems. This effect is also evident at lower temperatures (gel phase), where increasing concentrations of carotenoids are directly proportional to the wavenumber of the *ν*_s_CH_2_. The effect was more pronounced for the CL bilayers, reducing the T_m_ from 43.4 °C for the pure CL bilayers to 39.4 °C in the presence of 20% carotenoids. This effect might give evidence of a stronger alteration of lipid packing compared to DMPG, where T_m_ was reduced from 22.9 to 21.7 °C at the same concentration of carotenoids (Table 2). These results are in agreement with the results obtained by DSC.

The results obtained with the DMPG:CL mixture in the presence of different concentrations of carotenoids are presented in Figure 4C. The fluidization effect of the carotenoids observed in the one-lipid systems was not detectable for the 80:20 lipid mixture representative of *S. aureus*. However, the results were consistent with previous reports in the literature in which the presence of carotenoids was associated with a rigidification effect in the membrane [12]. Figure 4C showed that 1 to 5% of carotenoids induced an increase in T_m_, which is consistent with a more rigid membrane (Table 2). For higher concentrations of 10 and 20% carotenoids, a mild fluidization effect was observed on the DMPG:CL lipid system. The T_m_ of the mixture without carotenoids was 28.7 °C, and in the presence of 10 and 20% of carotenoids, the temperatures were shifted to 28.3 and 28.1 °C, respectively.

## 4. Discussion

In this work, the total carotenoid extract from the stationary phase of the methicillin-susceptible *S. aureus* strain was used to study the influence of carotenoids on the biophysical properties of the DMPG, CL, and DMPG:CL model membranes. The first step of this research was to obtain the total carotenoid extract of *S. aureus*. Marshall’s extraction protocol was adjusted to avoid degradation of carotenoids by incubation at 55 °C for 30 min, as foreseen in this method [32]. As a substitute, maceration with glass spheres and vortex was used for 5 min on pulverized cells (with a mortar and pestle), favoring the entry of the organic solvent by decreasing the particle size, to allow cell lysis [8]. The extracts then presented an intense orange color, associated with the presence of carotenoids. In addition, to prevent possible interferences generated by the peptides and proteins contained in this bacterial extract, secondary extraction with methanol:chloroform:water, resulting in elimination of these macromolecules, was performed. The composition of this carotenoid extract was previously reported. Early publications have focused on STX as an endpoint component of the biosynthesis of carotenoids in *S. aureus* [10,33]. However, we have previously reported that in the stationary growth phase, both STX and its precursor diaponeurosporenoic acid, which lacks glucose and fatty acid, were found in almost similar proportions [12]. In addition, it was found that there is a diversity of species of staphyloxanthin containing several saturated and branched fatty acids, indicating a variable composition of carotenoids in the native membrane state of *S. aureus*.

Carotenoids are not constitutively present in the membranes of *S. aureus* [12]. Instead, their synthesis is dependent on environmental triggers that are not yet fully understood, suggesting that carotenoid synthesis may be modulated to regulate the biophysical properties of *S. aureus* membranes based on need. This possibility motivates a search for a deeper understanding of the role of carotenoids in regulation of the thermotropic behavior of *S. aureus* membranes. For this purpose, we conducted a model study using two representative saturated phospholipid components of *S. aureus* membranes: DMPG and CL, and a mixture of DMPG and CL (ratio of 80:20 mol), representative of the cardiolipin content in the *S. aureus* membranes. This is a simplified model that does not take into account the presence of branched lipids, but is meant to explore the interrelation between phosphoglycerol and cardiolipin components. DSC and FTIR were used to investigate the effects of carotenoids on the thermotropic behavior of these mixtures with the aim of elucidating how carotenoids may influence the thermal phase behavior of the membrane in in vivo systems.

### 4.1. Effect of Carotenoid Content in Pure DMPG and CL Lipid Systems

The DSC results show that the addition of carotenoids induces significant changes in the thermotropic behavior of pure DMPG and CL systems. In the gel-phase temperature, the incorporation of carotenoids suppresses the L_β_/P_β_ transition for DMPG and the two cooperative events associated to the L_c_′/L_β_ transitions for CL [26]. This effect is likely associated with changes in the level of lipid-packing that may eliminate the ripple phase in the case of DMPG and the subtransition crystal phase in the case of CL. This is not an unusual observation when adding a second component (such as cholesterol or peptides) to these pure systems [34,35].

Focusing on the main melting event, the presence of carotenoids induces a reduction in the main cooperative gel and liquid–crystalline phase T_m_ for both DMPG (P_β_′ to L_α_) and CL (L_β_ to L_α_). The reduction in T_m_ is accompanied by a reduction in the transition enthalpy, in both cases indicating a smaller change in the molecular conformational energy associated with the phase transition. The reduction in T_m_ and transition enthalpy are very pronounced for CL, while less pronounced for DMPG. The reduction in T_m_ indicates that the presence of carotenoids strongly disrupts the gel phase of both pure lipid systems, being less disruptive to the liquid–crystalline phase. Transition enthalpy is mainly attributed to the increase in *gauche* rotamers in the acyl chains at the expense of *trans* rotamers and an associated increase in molecular spacing [28,36]. The observed reduction in the transition enthalpy owing to the presence of carotenoids most likely indicates a less pronounced jump in the *trans*/*gauche* ratio of the acyl chains between the two phases. This would indicate that the presence of carotenoids makes both phases more similar in relation to the *gauche*/*trans* rotamer ratio of the acyl chains.

The presence of carotenoids has been reported to increase acyl-chain order in *S. aureus* membranes at physiological temperatures, as measured by DPH anisotropy, where it is assumed that the membrane is in a liquid–crystalline phase [20,37,38,39]. DPH anisotropy findings have been complemented with studies using Laurdan generalized polarization and FT-IR on total lipid extracts of *S. aureus*, which indicate an increase in headgroup packing in the presence of carotenoids [11,40]. The general perception is that the presence of carotenoids increases the rigidity of the lipid bilayers in the *S. aureus* membranes under physiological conditions.

However, the present results give a broader picture of the effects of carotenoids in the *S. aureus* membranes. The depression of the phase-transition temperature indicates that carotenoids are very disruptive to the gel phase. This is in contrast to the classic results observed in the case of cholesterol in membranes, where cholesterol does not generate major changes in the phase-transition temperature of saturated phospholipids such as DPPC [41]. In the gel phase, cholesterol increases headgroup spacing [42] without increasing the level of *gauche* rotamers [43]. In contrast to cholesterol, the current results show that carotenoids are very disruptive to the gel phase.

The implications of this disrupted gel phase are important for *S. aureus* in relation to homoviscous regulation. *S. aureus* is found mainly on the skin and nose of human subjects, where it is exposed to variations in temperature and water content. Variations of both of these factors induce liquid–crystalline to gel phase transitions in phospholipid membranes, which can be deleterious for cells by causing loss of cell content [44]. Other bacteria, such as *Bacillus subtilis*, deal with variations in environmental temperature through the synthesis of unsaturated lipids [45,46,47], which is not an option for *S. aureus*. The depression of the main phase-transition event for DMPG and CL suggests that carotenoids may aid cells in maintaining a more fluid membrane at low temperatures, as also reported for *Staphylococcus xylosus* [40].

### 4.2. Effect of Carotenoid Content in DMPG/CL Mixtures

Cardiolipin has been reported to take part in the formation of lipid domains in bacterial membranes [48]. Little information has been reported regarding the role of cardiolipin in lipid domain formation in *S. aureus*. However, cardiolipin is known to accumulate in the stationary growth phase of this bacterium, reaching proportions of up to 20 mol% [49]. Carotenoids have been reported to regulate domain formation in *S. aureus*, and these lipid domains are associated with the pathogenicity of *S. aureus* [21]. In the second part of this study, we wanted to explore how carotenoids regulate the thermotropic melting behavior of 80:20 DMPG:CL mixtures, being representative of the phospholipid composition of *S. aureus*. The purpose of this investigation was to examine the effects of carotenoids in the range of the gel- to liquid–crystalline-phase coexistence regime, and the potential for regulation of lipid domain formation. The FT-IR and DSC results show a broad melting event for the 80:20 DMPG/CL mixture that spans from about 26 to 33 °C, which is expected in mixtures involving two lipids with differing melting temperatures. The presence of carotenoids clearly broadens the phase coexistence regime of this mixture. The broadening of the melting event strongly suggests that carotenoids expand the region for phase coexistence and domain formation, which is likely to influence domain formation.

### 4.3. Comparison with Live-Cell Systems and Other Model Systems

The results we obtained for model systems are consistent with observations from the in vivo analysis of *S. aureus* cell membranes. Phospholipid melting events have been identified in whole *S. aureus* cells [12] and total lipid extracts [11] by FT-IR and various fluorescence spectroscopy techniques. The main lipid melting event in *S. aureus* cells occurs in the range between 10 and 15 °C, with a smaller melting event that is consistently observed around 35 °C. When carotenoid synthesis is inhibited through knock-out of one of the enzymes in the synthesis pathway of staphyloxanthin, an increase in the temperature of the main melting event is observed [12]. Additionally, overexpression of carotenoids increases the CH_2_ stretch mode wavenumber at lower temperatures, indicating an increased level of disorder of acyl chains at low temperatures in the presence of carotenoids. A subtle difference of 0.5 °C was found between *S. aureus* cells that synthesize carotenoids and those that do not produce these pigments [12], comparable to 0.6 °C when 20% molar carotenoids were added to the DMPG:CL (80:20) system. Thus, the in vivo results could be considered as an experimental indication that the percentage of carotenoids in the bacterial membrane would be around 20% molar, and the tendency of these carotenoids is to decrease T_m_.

Similar results on the impact of STX homologues synthesized by *S. xylosus* on the biophysical behavior of this bacterium have been reported using fluorescence spectroscopy with the DPH probe in in vivo analyses [40]. *S. xylosus* exhibit significantly more fluid membranes at temperatures below 20 °C and more rigid (less fluid) membranes above 30 °C when carotenoids are synthesized. These results confirm FT-IR spectroscopy observations for *S. aureus* carotenoids [40]. Similarly, biophysical implications generated by zeaxanthin (a polar carotenoid) in the gram-negative bacterium *Pantoea sp.* YR343, which naturally synthesizes this carotenoid, have been reported [50]. Thus, carotenoids provide stabilization of the bacterial membrane during changes in temperature. Also, high-temperature tolerance were reported in carotenoid-producing *S. aureus* cells that were subjected to heat stress (58 °C) in the stationary phase of cell culture, compared to cells that do not produce carotenoids [22]. Carotenoids have been found to be synthesized in large amounts in bacteria found in Antarctica, and this overproduction has been associated with their ability to survive low temperatures [51].

Studies on liposomes containing zeaxanthin showed reduced membrane fluidity above the main phase-transition temperature, and increased fluidity below the transition temperature, by proton nuclear magnetic resonance [52] and electron paramagnetic resonance (EPR) [53]. Additionally, the influence of zeaxanthin on model 1,2-dihexadecanoyl-sn-glycero-3-phosphocholine (DPPC) liposomes was carried out using DSC [54]. The presence of 1% molar zeaxanthin in DPPC liposomes evidenced a decrease in molar heat capacity with a factor of two units and a broadening of the phase transition of the main peak observed in DSC [55,56].

## 5. Conclusions

The accumulated evidence of model systems and in vivo systems points to carotenoids playing an important role in homeoviscous response in bacterial systems by not only increasing membrane rigidity and mechanical resistance at physiological temperatures, but also maintaining fluidity at low temperatures. Also, the potential role of carotenoids in the regulation of lipid domains deserves careful exploration.

## Figures and Tables

**Table 1:** Phase-transition temperatures (T_m_), full-width at half-maximum (ΔT_1/2_), and peak height (Cp_max_) of deconvoluted peaks from representative DSC heating curves obtained for multilamellar liposomes prepared from DMPG and CL mixture (DMPG:CL, 80:20).

	Peak #1				Peak #2			
	T [°C]	ΔT_1/2_ [°C]	Cp_max_ [kJ mol^−1^ K^−1^]	ΔH [kJ mol^−1^]	T [°C]	ΔT_1/2_ [°C]	Cp_max_ [kJ mol^−1^ K^−1^]	ΔH [kJ mol^−1^]
DMPG:CL	28.10	3.33	4.41	16.88	31.27	2.38	4.00	11.12
+1 mol% Car	28.05	3.81	4.18	18.02	31.36	2.48	4.05	10.94
+3 mol% Car	27.61	4.38	3.95	19.45	31.10	2.76	3.77	11.15
+5 mol% Car	27.55	5.14	3.41	18.25	31.18	2.76	3.36	10.12
+10 mol% Car	27.18	5.71	3.05	18.85	31.13	2.86	3.23	10.98
+20 mol% Car	26.06	6.29	2.27	18.12	30.50	3.24	2.82	10.61

**Table 2:** Phase-transition temperatures (T_m_), of the fully hydrated supported bilayers of DMPG, CL, and DMPG:CL (80:20) by FT-IR. Standard deviations are ≤0.1 °C.

Lipids System/ Carotenoids (mol%)	DMPG	CL	DMPG:CL
0	22.9	43.4	28.7
1	22.6	42.0	30.1
3	22.5	41.1	29.3
5	22.4	40.9	29.4
10	21.7	40.9	28.3
20	21.7	39.4	28.1

## Supplementary Materials

The following are available at https://www.mdpi.com/article/10.3390/membranes12100945/s1. Table S1. Thermodynamic parameters of main phase transition and pretransition (#) of pure, fully hydrated DMPG multilamellar liposomes and DMPG/carotenoid mixtures determined from heating and cooling scans collected at a heating (cooling) rate of 1 °C min^−1^. Table S2. Thermodynamic parameters of phase transitions of pure, fully hydrated CL multilamellar liposomes and CL/carotenoid mixtures determined from heating and cooling scans collected at a heating (cooling) rate of 1 °C min^−1^.
