# Supplementary material for: Staphylococcus aureus Carotenoids Modulate the Thermotropic Phase Behavior of Model Systems That Mimic Its Membrane Composition
Source: Membranes (Basel). 2022 Sep 28;12(10):945. doi: 10.3390/membranes12100945 (PMC9612337; doi:10.3390/membranes12100945)
Supplement: Supplementary file 1 [file membranes-12-00945-s001.zip › membranes-1910910-supplementary.pdf]

Supplementary files

Table S1

Thermodynamic parameters of main phase transition and pretransition (#) of pure, fully hydrated DMPG multilamellar liposomes and DMPG/carotenoid mixtures determined from heating and cooling scans collected at a heating (cooling) rate of 1°C min^{-1}. The accuracy for the main phase transition temperature and enthalpy was ± 0.01°C and ± 0.8 kJ*mol^{-1}, respectively.

	Heating			Cooling		
	T [°C]	ΔH [kJ mol^{-1}]	ΔS [kJ mol^{-1} K^{-1}]	T [°C]	ΔH [kJ mol^{-1}]	ΔS [kJ mol^{-1} K^{-1}]
DMPG	23.86±0.06<br>15.03±0.07 (#)	29.80±0.42<br>3.64±0.26	0.100±0.006<br>0.013±0.002	23.84±0.02<br>14.69±0.06	31.11±0.52<br>12.39±0.48	0.105±0.008<br>0.043±0.004
+ 1 mol% Car	23.69±0.05<br>14.63±0.08 (#)	29.32±0.82<br>4.01±0.34	0.099±0.011<br>0.014±0.004	22.61±0.06<br>8.52+0.09	36.06±0.67<br>0.77±0.21	0.122±0.009<br>0.003±0.001
+ 3 mol% Car	22.42±0.04	23.90±0.65	0.081±0.009	21.22±0.04	25.89±0.42	0.088±0.008
+ 5 mol% Car	21.87± 0.06	21.99±0.56	0.075±0.008	20.62±0.05	24.90±0.63	0.085±0.006
+ 10 mol% Car	21.56±0.04	21.71±0.48	0.074±0.006	19.88±0.02	24.80±0.50	0.085±0.006
+ 20 mol% Car	22.02±0.08	21.46±0.74	0.073±0.008	19.74±0.07	24.87±0.89	0.085±0.009

Table S2

Thermodynamic parameters of phase transitions of pure, fully hydrated CL multilamellar liposomes and CL/carotenoid mixtures determined from heating and cooling scans collected at a heating (cooling) rate of 1°C min^{-1}. The accuracy for the main phase transition temperature and enthalpy was ± 0.01°C and ± 0.8 kJ* mol^{-1}, respectively.

	Heating			Cooling		
	T [°C]	ΔH [kJ mol^{-1}]	ΔS [kJ mol^{-1} K^{-1}]	T [°C]	ΔH [kJ mol^{-1}]	ΔS [kJ mol^{-1} K^{-1}]
CL	43.13±0.05	81.93±0.57	0.259±0.009	42.03±0.07	99.78±0.98	0.317±0.004
	28.45±0.06	12.46±0.23	0.040±0.002	-	-	-
	18.66±0.05	17.28±0.37	0.059±0.002	10.00±0.05	14.46±0.48	0.051±0.005
+ 1 mol% Car	42.87±0.08	94.96±0.87	0.291±0.008	41.72±0.06	118.18±1.24	0.375±0.008
	27.47±0.06	7.91±0.21	0.026±0.002	22.54±0.05	5.49±0.87	0.019±0.002
	17.82±0.07	21.57±0.62	0.074±0.003	8.62±0.07	9.04±0.79	0.032±0.004
+ 3 mol% Car	41.91±0.05	49.43±0.34	0.157±0.006	40.70±0.06	57.97±1.22	0.185±0.005
	24.61±0.08	2.48±0.12	0.008±0.002	-	-	-
	16.28±0.08	10.51±0.72	0.036±0.005	7.18±0.05	1.08±0.05	0.004±0.001
+ 5 mol% Car	41.36±0.04	44.77±0.45	0.142±0.006	40.08±0.07	63.14±0.92	0.216±0.004
	-	-	-	-	-	-
	16.78±0.06	7.06±0.11	0.024±0.003	-	-	-
+ 10 mol% Car	40.21±0.08	38.39±0.79	0.123±0.003	38.77±0.04	58.62±0.87	0.188±0.005
+ 20 mol% Car	37.88±0.09	29.29±0.36	0.094±0.006	36.13±0.03	46.93±0.73	0.152±0.007
	-	-	-	-	-	-
	-	-	-	-	-	-
	43.54+0.08	1.54±0.09	0.005±0.003	41.83±0.09	1.21+0.09	0.004±0.002
